# How the Spatial Position of Individuals Affects Their Influence on Swarms: A Numerical Comparison of Two Popular Swarm Dynamics Models

**DOI:** 10.1371/journal.pone.0058525

**Published:** 2013-03-21

**Authors:** Allison Kolpas, Michael Busch, Hong Li, Iain D. Couzin, Linda Petzold, Jeff Moehlis

**Affiliations:** 1 Department of Mathematics, West Chester University of Pennsylvania, West Chester, Pennsylvania, United States of America; 2 Department of Mechanical Engineering, University of California Santa Barbara, Santa Barbara, California, United States of America; 3 Department of Computer Science, University of California Santa Barbara, Santa Barbara, California, United States of America; 4 Department of Ecology and Evolutionary Biology, Princeton University, Princeton, New Jersey, United States of America; National Research & Technology Council, Argentina

## Abstract

Schools of fish and flocks of birds are examples of self-organized animal groups that arise through social interactions among individuals. We numerically study two individual-based models, which recent empirical studies have suggested to explain self-organized group animal behavior: (i) a zone-based model where the group communication topology is determined by finite interacting zones of repulsion, attraction, and orientation among individuals; and (ii) a model where the communication topology is described by Delaunay triangulation, which is defined by each individual's Voronoi neighbors. The models include a tunable parameter that controls an individual's relative weighting of attraction and alignment. We perform computational experiments to investigate how effectively simulated groups transfer information in the form of velocity when an individual is perturbed. A cross-correlation function is used to measure the sensitivity of groups to sudden perturbations in the heading of individual members. The results show how relative weighting of attraction and alignment, location of the perturbed individual, population size, and the communication topology affect group structure and response to perturbation. We find that in the Delaunay-based model an individual who is perturbed is capable of triggering a cascade of responses, ultimately leading to the group changing direction. This phenomenon has been seen in self-organized animal groups in both experiments and nature.

## Introduction

Many animal groups are self-organized, such as schools of fish [1], [2], flocks of birds [3], herds of wildebeest [4], and swarms of locusts [5]. These groups arise through social interactions among individuals, without necessitating centralized control or response to a template, or global cue [6]. Groups can be composed of many individuals, with each individual constantly interacting with its neighbors to maintain the collective motion. In this paper, we use the term *swarm* as a metaphor for a self-organized animal collective and develop generic models and theory which aim to have broad applicability. Swarms are believed to be maintained through simple positive and negative feedback mechanisms [6]. Individuals tend to repel from neighbors that are too close, and may be attracted to, or exhibit a tendency to align with, neighbors further away [2], [6]–[8]. Sensing and communication are critical to group formation and maintenance. For fish schooling, it is believed that vision and, for some species, the lateral line, an organ sensitive to changes in water pressure, are the main sensory systems involved [2], [9]. For flocking birds, vision and vocal communication are key [10]. Mathematical models have demonstrated that with a few simple behavioral interactions, mediated by sensing and communication, a variety of robust patterns of motion can emerge [1]. Typical collective patterns of motion that have been validated experimentally [11] include aggregates with cohesion but low levels of polarization, highly polarized mobile motion, and milling patterns in which the group rotates around an empty core [12]. Not only have models demonstrated the ability of swarms to switch between various collective patterns of motion in response to both changes in individual behaviors [12] and stochastic events [13], but these changes in collective behavior have also been shown to occur in real groups [11].

Group living may be advantageous to individuals, with benefits including increased foraging efficiency [2], [14], better ability to follow migration routes [15], [16], improved aerodynamic efficiency [3], [17], and a reduction in predation risk per group member [18], [19]. However, the costs and benefits of group membership are typically not evenly distributed among members [20]. For example, individuals located near the front of a fish school are more likely to maximize their food uptake but may have a greater risk of predation [21], while experimental data presented by Handegard et al. suggests that individuals at the rear of schools may generally be more vulnerable [22]. For birds flying in a Vee formation, it has been shown that the lead bird expends more energy than those trailing behind [23]. However, recent results for deformable bodies in flow suggest the opposite, namely that the leader of a group may benefit from a significant drag reduction in comparison to those trailing behind [24].

A primary advantage of living in groups is the ability to dynamically respond to changes in the environment such as migration routes, resources, or encounters with predators [8], [15], [25]. Highly polarized groups, such as flocks of birds and schools of fish, may benefit by acting as an array of sensors, facilitating the transfer of information to uniformed group members. Observations of natural fish schools as well as laboratory experiments have demonstrated that if a small number of individuals spot a predator or obstacle and abruptly change their direction of travel, this information, in the form of a rapid change in direction of travel (*heading*), can quickly propagate among members, allowing all individuals to escape [22], [26], [27]. Groups can tune their ability to respond to a stimulus by changing their structure [1]. For example, a highly polarized group may be more conducive to information transfer than a less polarized one [25].

**Figure 1:**
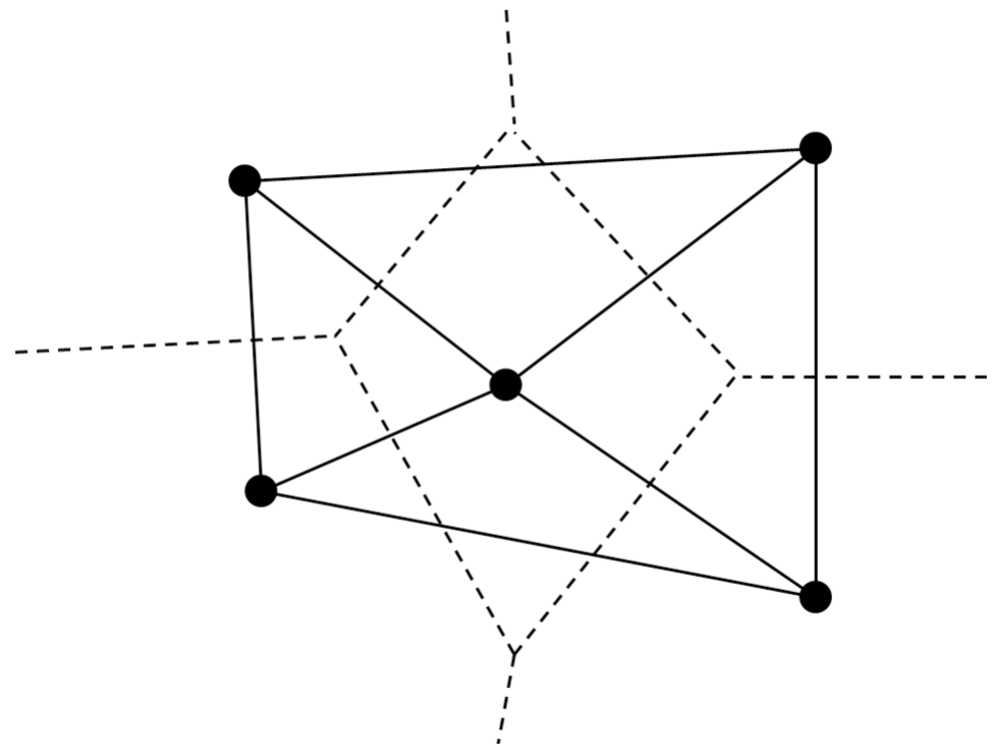
Relationship between a Voronoi Cell and its Delaunay Triangulation. The Voronoi cell associated with an individual in the swarm is the region of space which contains points that are closer to that individual than any other individual, where the boundary of a Voronoi cell (dashed lines) specifies two neighboring individuals. Black dots show the locations of the individuals. A Delaunay Triangulation defines triangular regions that are formed by drawing edges (solid lines) to connect nearest Voronoi neighbors. Therefore, the collection of edges given by the Delaunay Triangulation defines the swarm communication network topology when individuals within a swarm are constrained to communicate only with their nearest neighbors, as defined by Voronoi partitioning.

Although models of schooling have demonstrated how individual behaviors can lead to different collective patterns of motion, very few have studied their emergent internal dynamics. In this paper, we perform simulations to investigate the effect the communication topology has on the ability of a group to transfer information in the form of velocity information when perturbed. We consider two models: (1) a *zone-based* model which has a communication topology that relies on the intersecting regions of attraction, repulsion, and orientation among neighboring individuals, and (2) a *Delaunay-based* model where the communication topology of the swarm is determined by Delaunay Triangulation (see Figure 1). The models include a tunable parameter as in [15] which controls an individual's relative weighting of attraction and alignment. This parameter has a substantial impact on the geometric structure of the group and its ability to transfer information.

The computational experiments performed involve rapidly perturbing an individual's heading and measuring its influence by how much the swarm aligns with the disturbance. An individual's influence is directly related to its ability to transfer information and may vary with spatial position within the group. We compare the results of each model over different parameter values and population sizes. Our results show how relative weighting of attraction and alignment affect group structure and information transfer. Furthermore, they introduce previously unforseen benefits and drawbacks to adopting a particular spatial position within the group, which may have important consequences when considering real animal groups.

Individual-based models are a useful theoretical tool for investigating the dynamics of self-organized groups. However, the steady-state simulations required to accurately determine their statistical properties can be quite costly. To address this, simulations of individual-based models were parallelized both within and across realizations. In typical swarm models, individuals consider the relative positions and directions of neighbors, before updating their own position at each time step. This computation can be done more efficiently by parallelizing across individuals in the group. In addition, the replicate simulations needed to take statistics can be performed in parallel across realizations. Parallel simulations of the individual-based models studied were performed either on a multi-core computer or optimized for efficiency on a CUDA-enabled NVIDIA GPU. See Appendix S1 for more details on GPU computing.

### The Model

We consider a two-dimensional individual-based model for swarming in which interactions take place within two behavioral zones. This type of model was considered in [15], [28] with informed leaders. Here we assume there are no leaders and explore the effects of different weights of orientation and attraction response on the swarming behavior, and the relationship between spatial position and influence.

The zone-based model implicitly assumes that for small groups, an individual can monitor the states of each other member in the group. For larger groups, this assumption would become untenable because crowding restricts perception of others that are beyond immediate neighbors, and one should only consider the closest neighbors as stimuli [29]. Work by Ballerini et al. [30] suggests, from empirical evidence, that birds influence each other according to a communication topology similar to one derived from performing a Delaunay Triangulation of the flock. We will also explore this communication rule for 2-dimensional swarms, with emphasis on schools of fish.

### Zone-based Formulation

Groups are composed of 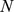 individuals with positions 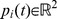 and unit directions 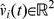. Individuals travel at constant speed *s* and have finite turning rate 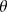. Every time step 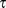, individuals simultaneously determine a new direction of travel by considering neighbors within two behavioral zones. The first zone, a “zone of repulsion”, is represented by a circle of radius 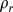 centered about the individual. Individuals repel away from others within their zone of repulsion. The second zone, a “zone of orientation and attraction”, is represented by an annulus of inner radius 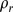 and outer radius 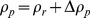 about the individual, excluding a blind area behind the individual, defined as a circular sector with interior angle 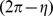 for which neighbors are undetectable. Individuals align with and are attracted towards neighbors within their zone of orientation and attraction. For a given individual *i*, let us denote the set of neighbors contained in the zone of repulsion as 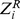, and the set of neighbors contained in the zone of orientation and attraction as 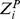. These zones are used to define the following behavioral rules of motion. If individual *i* finds other individuals within its zone of repulsion, then it orients its direction away from the average relative directions of those individuals. Its desired direction of travel in the next time step is given by the sum
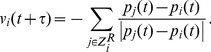
(1)


If individual *i* does not find other individuals within its zone of repulsion, then it aligns with (by averaging the directions of travel of itself and its neighbors) and feels an attraction towards (by orienting itself towards the average relative directions of) individuals within its zone of orientation and attraction. Its desired direction of travel is given by the weighted sum of two terms:
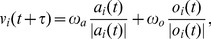
(2)where 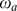 and 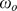 are the weightings of the attraction and orientation terms respectively, and
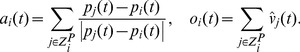
(3)The desired direction of travel of individual *i* is normalized as 
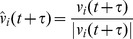
, assuming 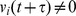. As before, if 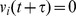, then individual *i* maintains its previous direction of travel as its desired direction of travel. We denote 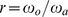 as the ratio of orientation and attraction tendencies.

To simulate movement errors, noise is added by rotating individual *i*'s desired direction 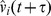 by an angle drawn from a circularly wrapped normal distribution with mean 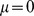 and standard deviation 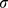. Also, since individuals can only turn 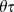 radians in one timestep, if the angle between 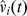 and 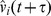 is greater than 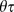, individuals do not achieve their desired direction, and instead rotate 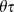 towards it. Finally, each individual's position is updated simultaneously as
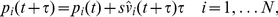
(4)where *S* is taken to be the constant speed of travel.

### Delaunay-based Formulation

In contrast to the zone-based model, local influence in animal swarms can be described by Voronoi partitions to define a nearest neighbor communication topology [30]. The communication topology determined by this framework is defined by the dual representation of the Voronoi partitioning of a space, otherwise known as a Delaunay Triangulation of that space [31] (see Figure 1). The rules of attraction and repulsion between neighboring individuals, in this case, are essentially the same as that of the *zone-based formulation* section, except that the the region of attraction is now unbounded.

For a finite collection of *N* individuals 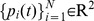, the Voronoi cell associated with the 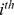 individual is 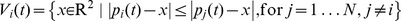, where 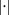 is the Euclidean norm [32]. Thus, by definition, two individuals, say 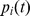 and 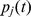, are said to be *neighbors* if and only if 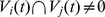; the points of intersection lie on the boundaries of the Voronoi cells. The Delaunay Triangulation of 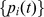 is then obtained by connecting each neighboring individual by a *Delaunay Edge*. Computing the state of the system for each time step is essentially the same as the zone-based model, except now for each individual, only neighbors who share a Delaunay Edge are included in each individual's local computation.

## Methods of Analysis

Two observables are used to measure the structure of the simulated swarms: elongation and polarization. Group elongation is computed by forming the minimal bounding box containing the group and taking the ratio of the length of the axis of the bounding box aligned with group motion to the axis perpendicular to group motion [15]. When a swarm is equally wide as it is long, 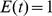. For our simulations, typically 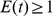. Polarization,
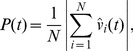
(5)measures the degree of group alignment. If all individuals within a swarm adopt the same heading, 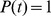, while if their headings balance out, 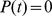. Thus, 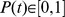. To obtain statistics regarding the group structure for a given set of parameters, one thousand steady-state simulations (with different initial conditions) were performed. At the beginning of each simulation, individuals are placed in a bounded region with randomized positions and directions of travel. The zone-based simulations were run in parallel on a graphics processing unit (GPU), while the Delaunay-based simulations were run in parallel on a multi-core computer. See [33] and Appendix S1 for more details on GPU computing. Simulations were run for 3000 timesteps to ensure the group had reached a steady collective pattern of motion, and the average group elongation and polarization were recorded as well as the probability of group fragmentation at steady-state. Furthermore, we use a timestep duration of 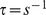 so that the dynamics are normalized with respect to the constant rate of speed of the individuals. A group is defined to be fragmented when it is composed of two or more non-interacting subgroups. In practice, an algorithm based on equivalence classes is used to determine the number of non-interacting subgroups.

The center of the group is defined as 
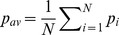
 and the average group heading is defined as 
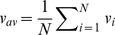
. To investigate the relationship between spatial position and individual influence we perform the following numerical experiments. We rotate the heading of a single individual by 90 degrees counterclockwise (with respect to their initial heading) and measure the correlation of the average heading of the group with the perturbed heading of the individual as a function of time. More specifically, define the cross-correlation function 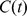 as
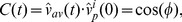
(6)where 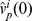 is the perturbed heading of individual *i* and 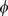 is the angle between 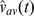 and 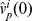. Thus, 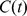 is a measure of the sensitivity of a group to a perturbation in the heading of an individual, and takes values in the range 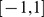. When 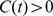, the group's heading has adjusted to be closer to the heading of the perturbed individual (positive correlation), while when 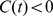, the group's heading has adjusted to be further away from the heading of the perturbed individual (negative correlation). This correlation function can be easily generalized to include the perturbation of multiple individuals (at a single time) by replacing the single perturbed heading in expression (6) with an average of the perturbed headings. To get statistics for 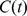, we average the results of these numerical experiments across the 1000 different initial conditions. First, we translate and rotate swarms so that they have the same center of mass 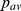 and average heading 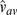. We then divide the plane into a lattice of spatial extent 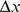 and average the results over each lattice point, discarding points with insufficient statistics (less than 5 individuals).

## Results

### Zone-based Results

#### Swarming Patterns

**Figure 2:**
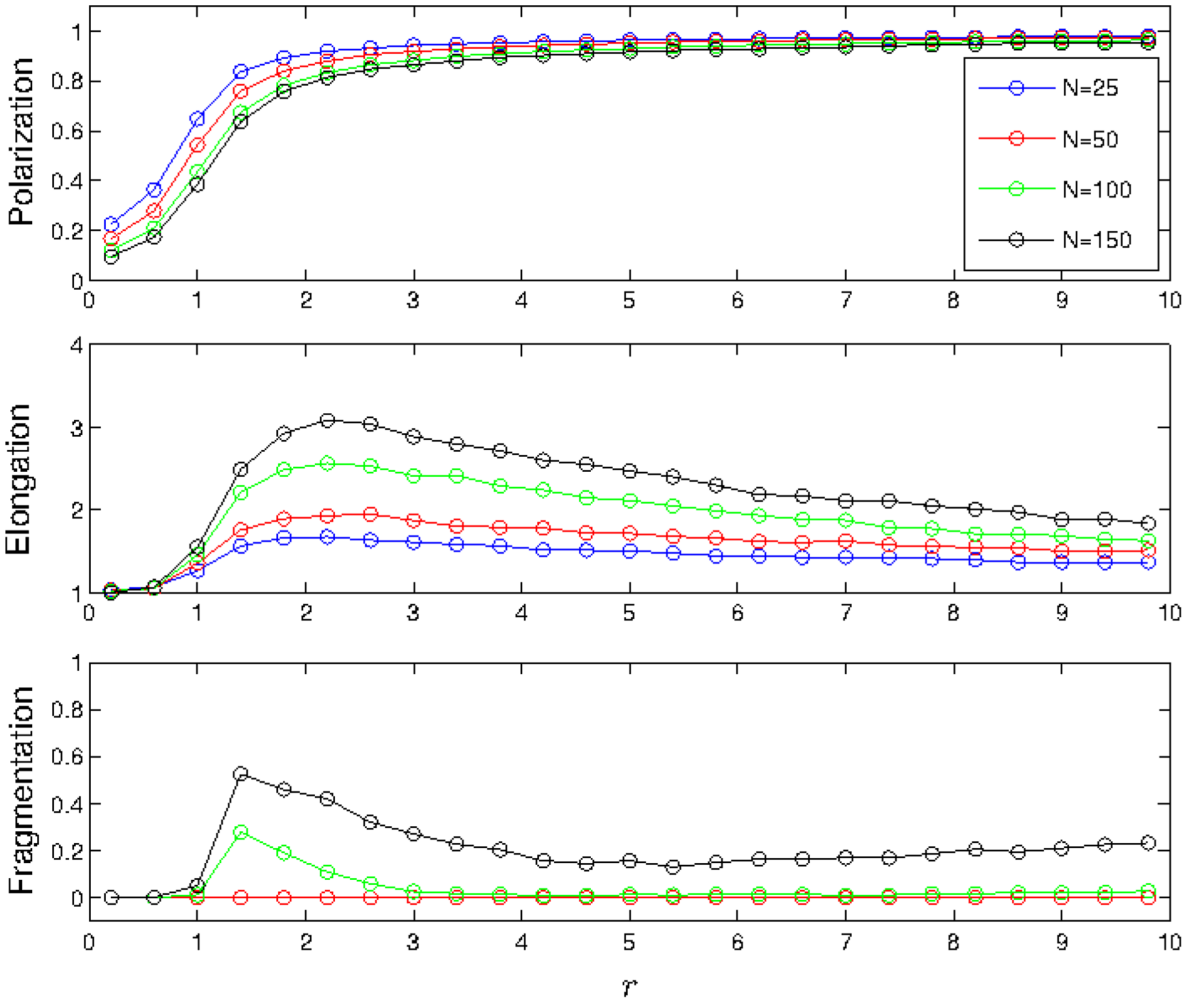
Average group polarization, elongation, and probability of fragmentation. Polarization, elongation, and probability of fragmentation are given as functions of *r*, the ratio of orientation to attraction weightings, for schools of size 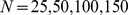 for the local zone-based schooling model. For schools of size 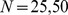, the probability of fragmentation is zero for all values of *r*. The standard deviation 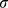 of polarization values is bounded by 0.20 for all values of *N*, while the standard deviation of elongation values are bounded by: 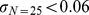, 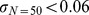, 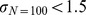, 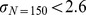.

As the ratio *r* of an individual's orientation to attraction tendencies is varied, different swarming patterns emerge. For *r* near zero, groups are cohesive with low levels of polarization. As *r* is increased, groups become more polarized, forming dynamically parallel and then highly parallel patterns of motion, using the terminology of [12]. For intermediate values of *r*, groups become elongated along their principle axis of motion. The probability of group fragmentation is correlated with both group polarization and elongation and does not simply increase with *r*; see Figure 2. In particular, we find that highly elongated groups with low levels of polarization are *more* likely to fragment than groups with higher levels of polarization. In [34] simulations were performed in the parameter regime of the local maxima of fragmentation and indicate that fragmentation arises in elongated groups as a pinching process from a narrow point in the group. When such an instability exists, fragmentation of the group into subgroups is likely. After this local maximum, fragmentation probability quickly decreases and eventually begins to slowly increase again, as a function of *r*. This is not surprising since highly polarized groups, where individuals weight alignment more heavily than attraction, splinter more frequently. This suggests that the trade-off between polarization and cohesion may not be as simple as previously expected [35]. In summary, our analysis demonstrates how by changing the relative weighting of orientation to attraction influences,individuals can influence the collective patterns of motion of a zone-based group.

#### Response to a Perturbation

**Figure 3:**
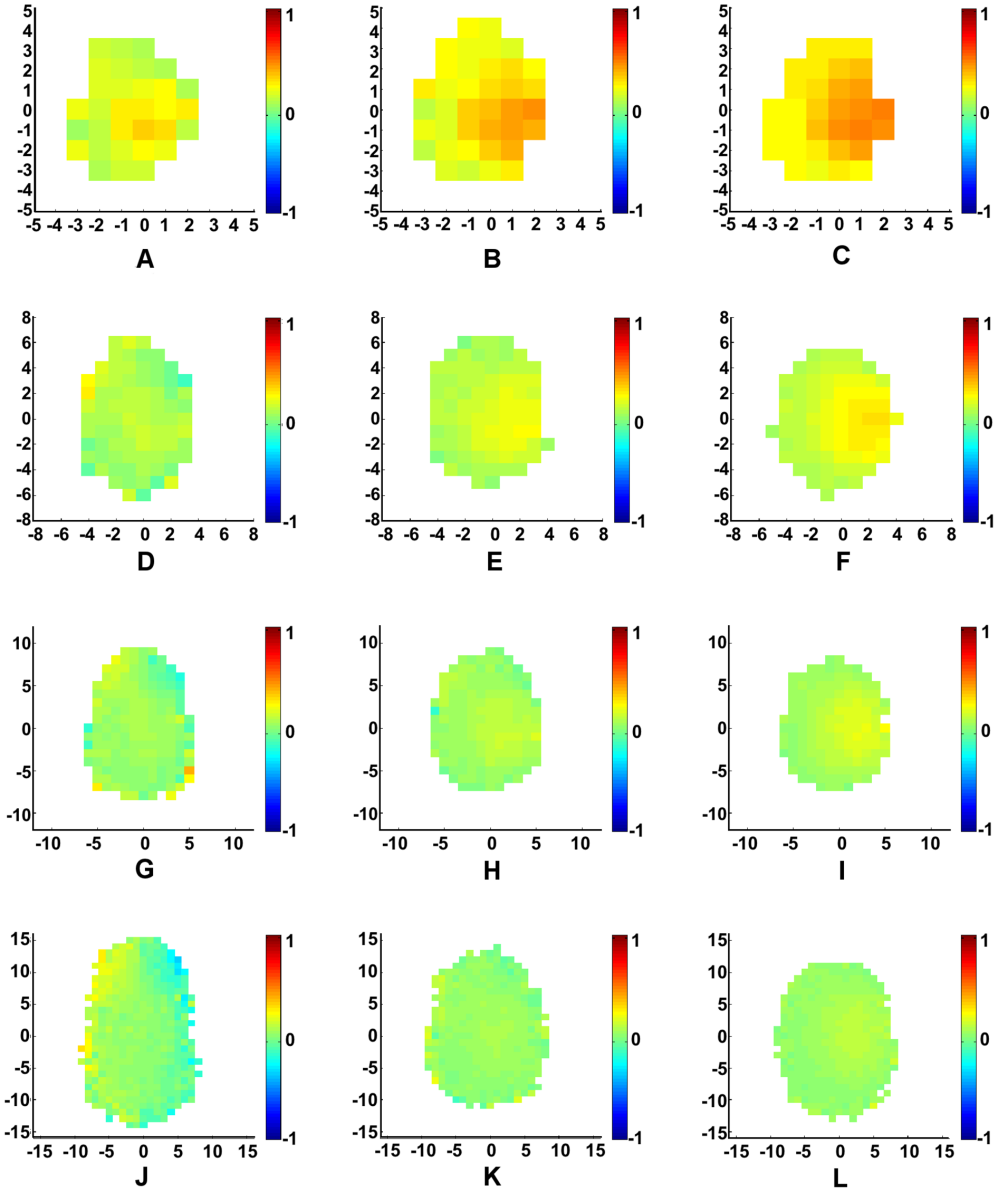
Average relative response of groups to a single perturbation for the zone-based model. (A) *N* = 10, *r* = 4, (B) *N* = 10, *r* = 16, (C) *N* = 10, *r* = 64, (D) *N* = 25, *r* = 4, (E) *N* = 25, *r* = 16, (F) *N* = 25, *r* = 64, (G) *N* = 50, *r* = 4, (H) *N* = 50, *r* = 16, (I) *N* = 50, *r* = 64, (J) *N* = 100, *r* = 4, (K) *N* = 100, Ì,, *r* = 16, (L) *N* = 100, *r* = 64. Results are colored according to 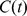, averaged over each point on a lattice of width 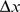 = 1 at time step 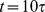. Groups are oriented so their center of mass is at the origin, and rotated such that the average direction of orientation aligns with the vertical axis. The perturbation was performed by rotating an individual counterclockwise by 90 degrees from the swarm's average direction of orientation. Boxes are only given a color if at least 5 individuals were averaged to compute that box's value. Standard deviation values 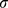 are bounded over all values of *N*, for each value of *r*: 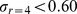, 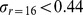, 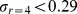.

The results of the perturbation analysis on the zone-based model depend on group size, polarization, and elongation; see Figure 3. As *r* is increased, small groups (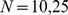) respond more strongly to internal perturbations, as measured by the cross-correlation function 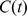. Thus, by changing local behavioral tendencies (adjusting *r*), individuals in small swarms can tune their collective sensitivity to fluctuations. For larger size schools (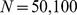) there is no substantial change in the level of response to a perturbation. The cross-correlation function is nearly zero for all positions so all individuals have negligible influence. This is not surprising since in the zone-based model, interactions are averaged over many individuals (see Table 1) so any fluctuation is quickly dampened for large enough groups.

**Table 1:** Average number of neighbors for each individual, as a percentage of total population in zone-based model.

*N*	*r* = 4	*r* = 16	*r* = 64
10	100	100	100
25	97.8	98.3	97.8
50	80.8	85.5	84.7
100	51.8	58.5	57.1

Our analysis also reveals some distinct spatial differences in the level of influence of individuals when perturbed. For very elongated groups with low levels of polarization (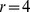, 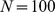), individuals turning away from the center of mass of the swarm have a slightly positive influence on the orientation of the swarm, while individuals who turn towards the swarm tend to have a slightly negative influence on the orientation of the swarm. In contrast, for small polarized groups (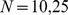, 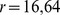), individuals turning toward the center of mass of the swarm have a large positive influence on its orientation, whereas individuals who turn away have a positive influence of lesser magnitude if any at all. There are no apparent trends in spatial differences in influence for all other parameters studied. It is noted that our results are completely symmetric about the turning angle. When individuals are perturbed clockwise (as opposed to counterclockwise), the spatial patterns of influence may be obtained by reflecting the results about the principal axis of motion of the group.

### Delaunay-based Results

#### Swarming Patterns

**Figure 4:**
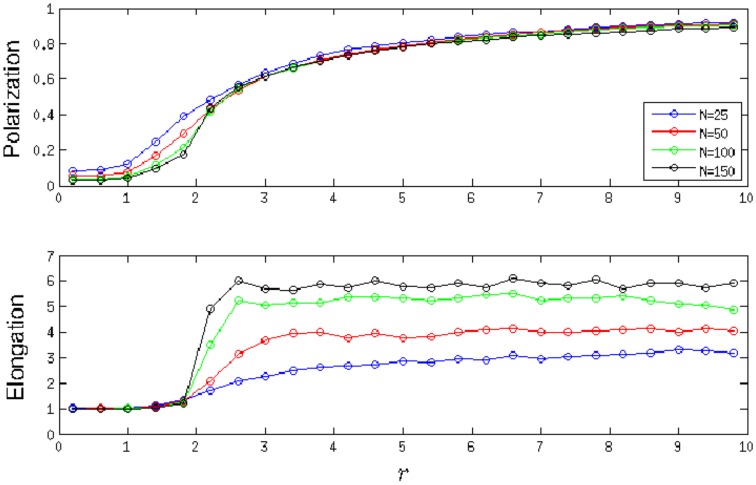
Average group polarization and elongation. Polarization and elongation are given as functions of *r*, the ratio of orientation to attraction weightings, for schools of size *N* = 25, 50, 100, 150 for the local Delaunay-based schooling model. The probability of fragmentation is zero for all values of *N* and *r*. For large values of *N*, a distinct phase transition occurs at 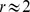, where the swarm becomes elongated and polarized. The standard deviation 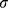 of polarization values is bounded by 0.16 for all values of *N*, while the standard deviation of elongation values are bounded by: 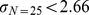, 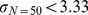, 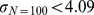, 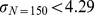.

**Figure 5:**
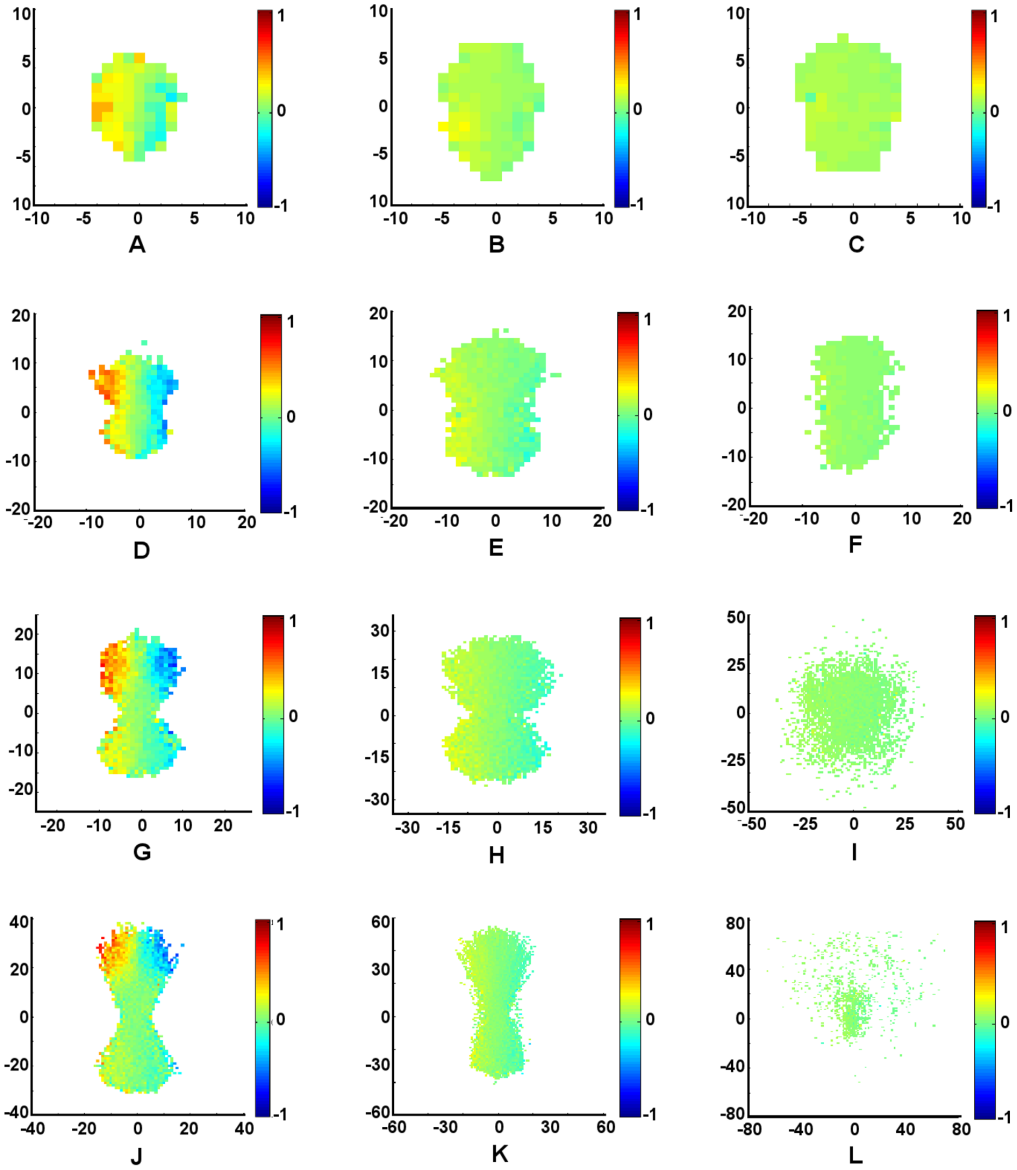
Average relative response of groups to a single perturbation for the Delaunay-based model. (A) *N* = 10, *r* = 4, (B) *N* = 10, *r* = 16, (C) *N* = 10, *r* = 64, (D) *N* = 25, *r* = 4, (E) *N* = 25, *r* = 16, (F) *N* = 25, *r* = 64, (G) *N* = 50, *r* = 4, (H) *N* = 50, *r* = 16, (I) *N* = 50, *r* = 64, (J) *N* = 100, *r* = 4, (K) *N* = 100, Ì,, *r* = 16, (L) *N* = 100, *r* = 64. Results are colored according to 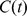, averaged over each point on a lattice of width 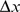 = 1 at time step 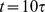. Groups are oriented so their center of mass is at the origin, and rotated such that the average direction of orientation aligns with the vertical axis. The perturbation was performed by rotating an individual counterclockwise by 90 degrees from the swarm's average direction of orientation. Boxes are only given a color if at least 5 individuals were averaged to compute that box's value. Standard deviation values 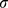 are bounded over all values of *N*, for each value of *r*: 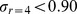, 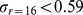, 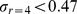.

**Figure 6:**
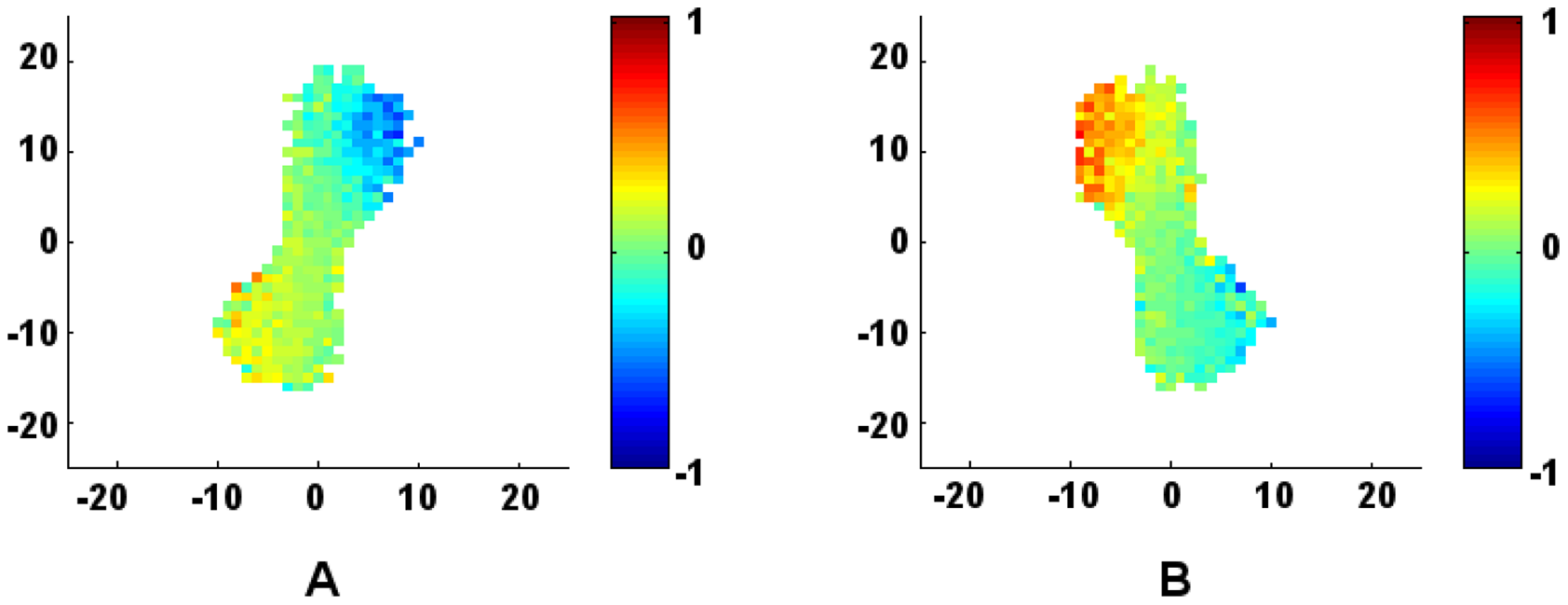
Symmetry of swarm influence. For the 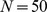 and 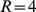 parameter values of the Dealunay-based perturbation results depicted in Figure 5(G), the collection of swarms are separated into (a) leftward aligned and (b) rightward aligned swarms. Averaging (a) and (b) produces Figure 5(G). Results are colored according to *C*, averaged over each point on a lattice of width 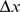 = 1 at time step 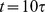.

When Delaunay Triangulation is used, communicating neighbors can be an arbitrary distance from each other. A consequence of this fact is that fragmentation is not allowed to occur without asserting additional constraints on how close individuals have to be to communicate, so we observed no fragmentation. Another consequence of this fact is that the instability of elongated schools in the zone-based model is not present in the Delaunay-based model. For *r* near zero, generally, groups are cohesive with low levels of polarization, and monotonically become more polarized as *r* is increased. Unlike the zone-based model, for large *r*, groups do not fragment but tend to be disperse. Although elongation of Delaunay-based swarms increases along with *r*, Figure 4 shows that elongation becomes more pronounced as the size of the swarm increases. It is noted that many of the swarm realizations for the Delaunay-based simulations tended to be aligned diagonally with respect to their heading. Here the individuals are not only elongated in their direction of travel, but also following at an angle of about 60 degrees from the individuals in front of them in many realizations, similar to the empirical findings of Katz et al. [36]. When averaging over all realizations, the skewed elongation of many realizations produces the hourglass formations observed in Figure 5 and Figure 6. As swarm size increases, for values of *r* that keep the swarm cohesive, the swarm continues to elongate along the diagonal of its elongation bounding box and leaves the aspect ratio of that bounding box relatively unchanged. Hence, we see in Figure 4 that the elongation values reach a certain point and then fluctuate slightly about that point.

#### Response to Perturbation

Both swarming patterns and response to perturbations are sensitive to parameter values when individuals are constrained to communicate only with their nearest neighbors. Figure 5 shows a general trend across most *r* values, where individuals turning away from the swarm have a positive influence on the orientation of the swarm, while individuals who turn towards the swarm tend to have a negative influence on the orientation of the swarm. For high values of *r*, we find that the swarm is spread out enough that no individual significantly influences the orientation of the swarm. Low values of *r* show a pronounced effect on the group that is biased towards the front of the swarm, and becomes more pronounced as *N* increases and the swarm becomes more elongated, as depicted in Figure 5(J).

As shown in Figure 5(L), we find that swarms with a relatively weak aggregate attractive force are able to slowly expand to sizes that would otherwise lead to fragmentation under the zone-based model. Hence, perturbation effects are more pronounced in Delaunay-based systems of lower *r*-value, where attractive forces are greatest. It is also noted that the elongated and skewed swarming patterns are more pronounced in the perturbation plots for low influence ratio, as seen in Figure 5. In this attraction-to-orientation regime, many of the swarm realizations tend to be aligned diagonally with respect to their heading. In this case, the influence of an individual turning either toward or away from the center of the swarm is observed more clearly in Figure 6, which shows that an individual's direction of perturbation with respect to the swarm center appears to have more of an influence on the swarm than its spatial location. It is noted that our results, again, are completely symmetric about the turning angle. When individuals are perturbed clockwise (as opposed to counterclockwise), the spatial patterns of influence may be obtained by reflecting the results about the principal axis of motion of the group.

## Discussion

By comparing Figure 3 and Figure 5, one sees that the effects of restricting interactions to nearest neighbors are significant. Whereas secondary and tertiary neighbors can be directly sensed if they are within an individual's region of attraction for the zone-based model, the secondary and tertiary neighbors can only influence an individual indirectly in the Delaunay-based model. For swarms with fewer than ten individuals, the communication topologies for the two models are very similar, and we would expect the two systems to behave most similarly in this regime. The swarm formation geometry of the Delaunay perturbation plots for the 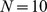 regime most similarly resemble that of the zone perturbation plots, even though the influence behavior is quite opposite. Specifically, for 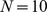, the most influential individuals of zone-based model turn towards the swarm center, while the most influential individuals of the Delaunay-based model turn away. When comparing Figure 2 to Figure 4, it seems that Delaunay-based swarms become geometrically different from zone-based swarms as *r* increases, according to their elongation and polarization coarse descriptions. For all values of *N*, swarm elongation is more pronounced for the Delaunay-based model and appears to quickly plateau, while swarm elongation of the zone-based model increases to a peak value near 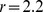 and then proceeds to monotonically decrease. Swarm polarization seems to monotonically increase for both models, but the increase seems to occur at a faster rate for the zone-based model.

When comparing Figures 3 and 5, it is observed that the overall influence of each individual is less for the zone-based model than for the Delaunay-based model. Because fewer individuals are included in the averaging algorithm for Delaunay-based swarms, each individual neighbor has a greater proportion of influence. Since the Delaunay-based swarms are more elongated, yet have greater influence across the swarm when compared to the zone-based results of similar parameter values, the effects of perturbations on the larger Delaunay-based swarms tend to cascade through the system. With the exception of ratio value 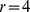 and population size 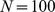, an important difference between the zone-based and Delaunay-based models is that the zone-based model shows greatest influence on the swarm from individuals who turn towards the swarm center, while the Delaunay-based model shows the opposite behavior. It is only for the 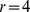 and 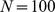 regime where the two models appear to agree in terms of regions of influence, and suggests that the strength of “turning away” leader behavior emerges as a result of cascading phenomena. As more individuals are introduced to the zone-based swarm, the average number of neighbors as a percentage of the total population decreases in value (see Table 1), which indicates that the information propagation mechanisms of the zone-based system become more cascade-like, as in the Delaunay-based system. It is also apparent that for elongated cohesive groups in the Delaunay-based model (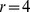 and 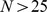), individuals at the front of the swarm have a more dramatic impact than those located towards the rear. This suggests that information propagates from the front to the back.

Moreover, unlike the zone-based model where the communication topology is related to the density of the swarm (the average number of neighbors in an arbitrary individual's zone of communication increases with swarm density) the average number of neighbors for each individual under Delaunay Triangulation is independent of swarm density since only nearest neighbors are considered. This nearest neighbor versus density phenomenon is summarized in Tables 1, 2, 3, and 4, which correspond with the data of Figures 3 and 5. It is noted that the average number of neighbors each individual can have under Delaunay Triangulation is upper bounded by 6 [31].

**Table 2:** Average number of neighbors for each individual, as a percentage of total population in Delaunay-based model.

*N*	*r* = 4	*r* = 16	*r* = 64
10	47.2	48.1	48.3
25	21.5	21.7	21.4
50	11.4	11.4	10.4
100	5.81	5.81	5.19

**Table 3:** Zone-based model swarm density (individuals/area).

*N*	*r* = 4	*r* = 16	*r* = 64
10	0.981	0.848	0.884
25	0.647	0.646	0.639
50	0.526	0.559	0.576
100	0.144	0.0144	0.00525

The density of a swarm can be determined by dividing the number of individuals in the swarm by the area of the bounding box that encloses the swarm, which is the same bounding box used to compute swarm elongation.

**Table 4:** Delaunay-based model swarm density (individuals/area).

*N*	*r* = 4	*r* = 16	*r* = 64
10	0.493	0.357	0.317
25	0.238	0.143	0.129
50	0.0457	0.0717	0.00864
100	0.0320	0.0633	0.00157

The density of a swarm can be determined by dividing the number of individuals in the swarm by the area of the bounding box that encloses the swarm, which is the same bounding box used to compute swarm elongation.

We find that under the zone-based model, swarm density tends to decrease more with respect to increases in swarm population than it does for increases in the ratio *r*, as shown in Table 3. We also find from Table 1 that the average number of neighbors belonging to any individual increases with swarm population size, and is irrespective of the ratio *r*. For the Delaunay-based model, Table 4 shows that swarm density decreases as the ratio *r* increases, and decreases as population size increases. Unlike what we observed for the zone-based system, the Delaunay-based system consistently produces between 4 and 6 neighbors per individual on average, as inferred from Table 2.

### Conclusions

The results demonstrate how increasing or decreasing the relative strength of orientation to attraction between an individual and its neighbors can affect an individual's influence on the group's behavior. For both models, groups become more polarized as individuals weight orientation more heavily. However, this comes at a cost with higher levels of fragmentation in the zone-based model and large spatial spread in the Delaunay-based model.

Our perturbation analysis shows that an individual is most influential when its effects are able to cascade from the front to the back of the swarm. In the low orientation to attraction regime (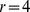) of the Delaunay-based model, groups are highly elongated and have suffiently large levels of polarization so that they are able to effectively propagate information in this manner. In the zone-based model we did not see this phenomena but the results indicate that this may be the case if groups are much larger than those studied. However, we do find that for small group sizes (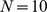) in the zone-based model, individuals can have a relatively large influence by “pushing” towards the swarm's centroid in contrast to the more typical influence by “pulling” away from the swarm's centroid seen in the Delaunay-based model.

The perturbation analysis also helps explain the diagonal structure of the swarms in the Delaunay-based model for low values of *r* where attractive forces are strong enough to keep members close enough to have meaningful communication. As a swarm continues to move forward, it elongates along its average direction of travel. In addition, as seen from the perturbation analysis, groups are most responsive to an individual's motion in the direction away from the swarm's centroid which leads to horizontal elongation. The combination of these two factors could explain why equilibrium swarm formations become diagonally elongated in the Delaunay-based model. In addition, the arctangent of the Elongation from Figure 4 gives the average following angle of the swarm for each population size, and the trend indicates that following angle increases with population size.

Some empirical evidence has shown the zone-based model to be an ample descriptor of swarm behavior for relatively small groups (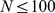), while other empirical studies have suggested that a Delaunay-based model may be more appropriate for swarms that are greater in number (up to at least 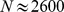) and more spatially spread out [30]. Our numerical study has shown distinct differences between the emergent behavior of the two models even for relatively small groups which may have not been identified if the swarm was solely classified using observables such as polarization and elongation. Our analysis provides a finer measure of the dynamics of a swarm by testing its response to perturbations from individual members as a function of spatial position. Such perturbations can occur frequently in swarms where a few individuals respond to a nearby external stimulus such as a predator or food source triggering a cascade of responses ultimately leading to the group changing its direction of motion [26], [27].

## Supporting Information

Appendix S1 **Parallel Simulation on a GPU.** Many of the simulations of the model were run in parallel on a graphics processing unit (GPU). A discussion of the implementation is included in Appendix S1.
(PDF)
