# Supplementary material for: How the Spatial Position of Individuals Affects Their Influence on Swarms: A Numerical Comparison of Two Popular Swarm Dynamics Models
Source: PLoS One. 2013 Mar 21;8(3):e58525. doi: 10.1371/journal.pone.0058525 (PMC3605438; doi:10.1371/journal.pone.0058525)
Supplement: Appendix S1 — Parallel Simulation on a GPU. Many of the simulations of the model were run in parallel on a graphics processing unit (GPU). A discussion of the implementation is included in Appendix S1. (PDF) [file pone.0058525.s001.pdf]

Appendix S1

To accurately determine the statistical properties of the collective motion of groups whose dynamics are described by such a model, many steady-state simulations are required. Simulations of the individual-based model can be quite costly and can benefit from parallel processing. In the model, each individual updates its direction of travel based on the positions and directions of travel of all other individuals. This computation can be performed in parallel across individuals within a single realization. In addition, replicate steady-state simulations can be performed in parallel across realizations. Both are data-parallel computations in which the same instructions are executed on multiple data elements in parallel.

A Graphics Processing Unit (GPU) is an inexpensive yet powerful alternative to a cluster for parallel simulations. Its architecture is very well suited to simulations of the individual-based schooling model. Parallel computation on general-purpose GPUs (GPGPU) has become an active research area with a wide range of scientific applications including fluid dynamics, cellular automata, particle systems, and neural networks [1–3]. Previous generation GPUs have required computations to be recast into a graphics applications programming interface such as OpenGL, which has made programming GPUs for non-graphics applications a significant challenge. In 2007, NVIDIA [4] released a Compute Unified Device Architecture (CUDA), a new hardware and software architecture for issuing and managing data-parallel computations on the GPU [5]. The CUDA API is an extension of the C programming language, which results in a minimal learning curve for beginners to access the low-level hardware.

To perform parallel simulations of the schooling model, we used a CUDA-enabled NVIDIA GeForce 8800GTX chip with 768 MB RAM installed on a host workstation with Intel Pentium 3.00 GHz CPU and 3.50GB of RAM with physical address extension. The chip has 128 stream processors, divided into 16 clusters of multiprocessors with eight streaming processors per multiprocessor. The eight processors in each multiprocessor share 16K shared memory, bringing data closer to the arithmetic logic unit (ALU). The global memory adjacent to the GPU chip is much larger, but has a much higher latency than the on-chip shared memory. It takes about 400-600 clock cycles to read or write to the global memory vs. 4 clock cycles to access the shared memory. The GPU chip only supports single-precision operations, which is sufficient for our schooling simulations. Individual GPU program launches can run at most 5 seconds on a GPU with a display attached, which is an artifact of its original function as a graphics rendering device.

The GPU is still a specialized device. It can only support single-precision operations and is only very efficient for applications with high computation per memory access and single instruction multiple data computation [6]. To efficiently make use of the GPU's computational resources, one must maximize the number of threads running in parallel, while taking the limited shared memory size into account. By a thread, we mean an executable task on a set of data. Although it is not difficult to migrate code to a GPU, careful implementation is necessary for good performance.

We programmed the GPU to perform multiple steady-state simulations of the swarming model, parallelizing within a single realization and across multiple realizations. To effectively use the GPU, we make as much use of the on-chip shared memory as possible. For parallelization within a realization, there is a limit to the number of individuals that can be stored in shared memory. We thus perform behavioral influence computations in parallel for n individuals at a time, where the group is divided into m groups of n individuals. In addition to parallel processing within a single realization, multiple independent replicate simulations are performed in parallel. Whenever a thread is waiting to access the device memory (e.g., to load the current position and heading of individual i), another thread is running on the ALUs (e.g., to compute the desired heading of individual j). Thus, memory access latency is not a problem. To generate random numbers, which are needed to generate initial conditions and to add noise to our calculations at each step, we employed a modified version of Eric Mills' multi-threaded C implementation of the Mersenne Twister (MT) algorithm [7]. For more details on the parallel implementation of the model, see [8]. For another successful approach to accelerating simulations of a swarming model on a GPU, see [9].

We observed speedups of 230-240 times for our parallelized code running on the GPU over the corresponding sequential simulation on the host workstation. It takes only a few minutes to perform 1000 steady-state simulations (lasting 3000 steps) of the model for swarms of size $N = 100$ over a mesh of 25 different values of the parameter r. The corresponding serial simulation on the CPU takes almost an entire day to complete.

References

1. GPGPU-Home (2012). GPGPU homepage. http://www.gpgpu.org/.

2. Owens JD, Luebke D, Govindaraju N, Harris M, Krger J, et al. (2005) A survey of general-purpose computation on graphics hardware. In: Eurographics 2005, State of the Art Reports. pp. 21–51.

3. H Li and L Petzold (2009) Efficient parallelization of the stochastic simulation algorithm for chemically reacting systems on the graphics processing unit. Int J of High Performance Computing Applications 24: 107–116.

4. NVIDIA Corporation (2012). NVIDIA homepage. http://www.nvidia.com.

5. NVIDIA Corporation (2012). Cuda C programming guide. http://developer.nvidia.com/nvidia-gpu-computing-documentation.

6. W-M Hwu (2007) GPU computing–programming, performance, and scalability. In: Proceedings of the Block Island Workshop on Cooperative Control.

7. NVIDIA Forums members (2012). NVIDIA forums. http://forums.nvidia.com.

8. Li H, Kolpas A, Petzold L, Moehlis J (2009) Parallel simulation for a fish schooling model on a general-purpose graphics processing unit. Concurr Comput : Pract Exper 21: 725–737.

9. Erra U, Frola B, Scarano V, Couzin ID (2009) An efficient GPU implementation for large scale individual-based simulation of collective behavior. In: HIBI '09 Proceedings of the 2009 International Workshop on High Performance Computational Systems Biology, IEEE Computer Society. pp. 51-58.
